# Global Research Landscape on Leptin Resistance and Obesity‐Related Metabolic Dysfunction: Trends, Networks and Visualisation Analysis (1995–2025)

**DOI:** 10.1002/edm2.70330

**Published:** 2026-08-27

**Authors:** Sa'ed. H. Zyoud

**Affiliations:** ^1^ Department of Clinical and Community Pharmacy, Faculty of Pharmacy An‐Najah National University Nablus Palestine; ^2^ Clinical Research Centre An‐Najah National University Hospital Nablus Palestine

**Keywords:** bibliometrics, cardiometabolic risk, inflammation, insulin resistance, leptin resistance, metabolic dysfunction, obesity, Scopus, VOSviewer

## Abstract

**Background:**

Leptin resistance is closely linked to obesity, insulin resistance, inflammation and cardiometabolic disorders. However, the global research output on leptin resistance and obesity‐related metabolic dysfunction has not been mapped in detail. This study aimed to analyse global research trends in this field from 1995 to 2025.

**Methods:**

Publications were retrieved from the Scopus database. Bibliometric indicators included annual growth, document type, productive countries, institutions, journals, highly cited publications and international collaboration. VOSviewer version 1.6.21 was used to visualise country collaboration and title–abstract term co‐occurrence.

**Results:**

A total of 2162 publications were included. Publication output increased substantially across the study period but fluctuated, peaking in 2014 and remaining broadly stable thereafter. Articles were the most common document type (*n* = 1658; 76.69%), followed by reviews (*n* = 433; 20.03%). The United States ranked first (*n* = 741; 34.27%), followed by China (*n* = 255; 11.79%), Japan (*n* = 132; 6.11%), Spain (*n* = 131; 6.06%) and Germany (*n* = 124; 5.74%). Harvard Medical School was the most productive institution, and Endocrinology was the most active journal. Cowley et al. had the most cited publication, with 2052 citations. Term mapping revealed three main themes: ‘clinical metabolic dysfunction and cardiometabolic risk,’ ‘hypothalamic leptin signalling and energy homeostasis,’ and ‘body weight regulation and developmental programming.’

**Conclusions:**

Research on leptin resistance and obesity‐related metabolic dysfunction expanded substantially between 1995 and 2025, although annual output fluctuated and plateaued after its 2014 peak. The maps suggest a broadening of terminology from early leptin biology and experimental models towards inflammatory, cardiometabolic and mechanistic topics; this pattern should not be interpreted as proof of a causal or complete transition. Collaboration remained concentrated among high‐output countries.

## Graphical abstract

Analysis of 2162 Scopus publications showed that research on leptin resistance and obesity‐related metabolic dysfunction expanded markedly from 1995 to 2025, peaking in 2014 before stabilising. The field broadened towards inflammatory, cardiometabolic and mechanistic topics, while international collaboration remained concentrated among high‐output countries.

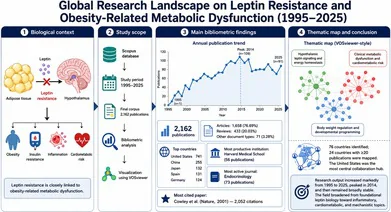

## 1 Introduction

Obesity is among the most important public health problems worldwide, not only because of excess body weight but also because of its close relationship with several metabolic and cardiovascular complications. Obesity‐related metabolic dysfunction includes insulin resistance, Type 2 diabetes, hypertension, fatty liver disease, chronic low‐grade inflammation and cardiovascular disease [1, 2]. These conditions are usually interconnected through complex endocrine, inflammatory, neuronal and metabolic pathways. In this context, leptin has attracted considerable scientific attention because it plays important roles in appetite regulation, energy expenditure, adiposity, glucose homeostasis and body weight control [3, 4].

Leptin is an adipocyte‐derived hormone that reflects body fat stores and regulates energy balance mainly through central hypothalamic pathways. Early studies have shown that leptin levels are closely related to body lipid content and adiposity, but many individuals with obesity have high leptin concentrations without an adequate biological response [5, 6]. This condition, known as leptin resistance, has become a key concept in explaining why increased adiposity does not necessarily translate into effective appetite suppression or weight reduction. Experimental studies have further shown that leptin activates anorexigenic pro‐opiomelanocortin neurons in the arcuate nucleus and influences hypothalamic neuropeptide pathways involved in food intake, glucose regulation and body weight control [7, 8, 9].

Over time, the understanding of leptin resistance has moved beyond simple measurement of circulating leptin levels towards a broader mechanistic framework. Several mechanisms, including impaired leptin transport, altered receptor signalling, suppressor of cytokine signalling 3 (SOCS3) activation/upregulation, protein tyrosine phosphatase 1B activity, hypothalamic inflammation and endoplasmic reticulum stress, have been proposed [10, 11, 12, 13, 14]. Moreover, leptin resistance has been increasingly linked to obesity‐related metabolic dysfunction, including changes in adipose tissue endocrine activity, insulin resistance, the gut microbiota, low‐grade inflammation, Type 2 diabetes, obesity‐associated hypertension and cardiovascular complications [15, 16, 17, 18, 19, 20]. Recent reviews further support the role of leptin resistance as a bridge between obesity, impaired metabolic regulation, cardiometabolic disease and potential therapeutic challenges [19, 20, 21].

These findings further indicate that leptin resistance is not only a disorder of appetite regulation but also part of a wider metabolic and cardiometabolic network [15, 16, 17, 18, 19, 20].

Despite the growing number of publications in this field, the global research landscape on leptin resistance and obesity‐related metabolic dysfunction has not been comprehensively mapped. It remains unclear how scientific output has changed over time; which countries, institutions, journals and publications have contributed most to the field; and which research themes have emerged as dominant or more recent topics. A bibliometric approach is useful for answering these questions because it can quantify research productivity, identify influential studies and contributors, visualise collaboration patterns and detect the thematic evolution of a body of literature.

Therefore, this study aimed to map the global research landscape on leptin resistance and obesity‐related metabolic dysfunction from 1995 to 2025 using bibliometric and visualisation methods. Specifically, the study examined annual publication trends, document types, leading countries, productive institutions, active journals, highly cited publications, international collaboration networks and major thematic clusters on the basis of title and abstract term co‐occurrence. This analysis may help to organise the existing evidence, identify research gaps and guide future experimental, clinical and translational studies in this expanding field.

Beyond descriptive ranking, the study addressed three questions: whether publication activity showed continued growth or a later plateau; whether productivity, citation influence and collaboration were concentrated among a limited set of contributors; and whether the vocabulary of the field broadened over time from central leptin biology towards metabolic and clinical topics. Temporal term patterns were treated as descriptive signals that may also reflect changes in terminology and indexing.

## 2 Methods

### 2.1 Study Design

This study was designed as a bibliometric and visualisation analysis of global scientific publications on leptin resistance and obesity‐related metabolic dysfunction. The methodological approach was based on previously published bibliometric studies that used Scopus data to map global research trends, scientific productivity, citation patterns and visualisation networks in related biomedical fields [22, 23, 24, 25]. Bibliometric methods are useful for evaluating the development of a research field, identifying influential publications and contributors and detecting hot topics and emerging research directions [26, 27].

### 2.2 Data Source

The Scopus database was used as the source of the bibliographic data. Scopus was selected because it is one of the largest abstract and citation databases of peer‐reviewed literature and provides broad coverage of biomedical, clinical and multidisciplinary journals. It also allows the export of bibliographic information needed for citation analysis and visualisation mapping, including authors, titles, abstracts, keywords, affiliations, countries, sources, document types, citation counts and references. Similar Scopus‐based approaches have been used in previous bibliometric studies on nonalcoholic fatty liver disease, insulin resistance, binge drinking and the gut microbiome [22, 23, 24, 25]. The final search was conducted on August 9, 2026. Because Scopus records and citation counts are continuously updated, all bibliometric indicators and citation data reported in this study reflect the status of the database on that date.

### 2.3 Search Strategy

The search strategy was developed to retrieve publications related to leptin resistance and obesity‐related metabolic dysfunction. The search was conducted in Scopus using terms related to leptin resistance, obesity, insulin resistance, metabolic syndrome, metabolic dysfunction, diabetes, inflammation and cardiometabolic risk. The search was performed in the title and abstract fields to improve the specificity of the retrieved documents. The following search query was used: TITLE‐ABS((‘leptin resistance’ OR ‘central leptin resistance’ OR ‘peripheral leptin resistance’ OR ‘resistance to leptin’ OR ‘leptin insensitivity’ OR ‘leptin signaling impairment’ OR ‘impaired leptin signaling’ OR ‘leptin signalling impairment’ OR ‘impaired leptin signalling’ OR ‘leptin receptor resistance’ OR ‘leptin receptor dysfunction’ OR ‘leptin pathway dysfunction’ OR ‘leptin resistance mechanism*’ OR ‘leptin sensitivity’ OR ‘leptin responsiveness’) AND (obes* OR overweight OR adiposity OR ‘body mass index’ OR BMI OR ‘visceral adiposity’ OR ‘abdominal obesity’ OR ‘metabolic dysfunction’ OR ‘metabolic syndrome’ OR ‘insulin resistance’ OR ‘type 2 diabetes’ OR T2DM OR ‘glucose intolerance’ OR ‘hyperinsulinemia’ OR ‘dyslipidemia’ OR ‘fatty liver’ OR ‘nonalcoholic fatty liver disease’ OR NAFLD OR ‘metabolic associated fatty liver disease’ OR MAFLD OR ‘metabolic dysfunction‐associated steatotic liver disease’ OR MASLD OR inflammation OR ‘low‐grade inflammation’ OR ‘adipose tissue dysfunction’ OR ‘energy homeostasis’ OR ‘appetite regulation’)) AND PUBYEAR > 1994 AND PUBYEAR < 2026 AND (LIMIT‐TO (SRCTYPE, ‘j’)) AND (EXCLUDE (DOCTYPE, ‘tb’) OR EXCLUDE (DOCTYPE, ‘er’)).

The expressions ‘leptin sensitivity’ and ‘leptin responsiveness’ were included because relevant experimental and translational studies may describe impaired biological response without using the exact phrase ‘leptin resistance.’ These broader expressions were not used alone; they were combined with the obesity and metabolic‐dysfunction concept block. To evaluate the effect of these expressions on the corpus boundary, the Scopus search was rerun on August 10, 2026 after removing only ‘leptin sensitivity’ and ‘leptin responsiveness,’ while retaining all other terms, publication‐year limits, source‐type restrictions and document‐type exclusions. Records were compared using unique Scopus EIDs.

The search was limited to documents published between 1995 and 2025. No language restriction was applied during the initial search. The search date was recorded to ensure reproducibility of the retrieved dataset.

### 2.4 Eligibility Criteria

The retrieved documents were limited to publications related to leptin resistance and obesity‐related metabolic dysfunction. Original articles, reviews, conference papers, short surveys, notes and letters were included. Documents that were not relevant to the study topic or did not clearly address leptin resistance in the context of obesity‐related metabolic dysfunction were excluded. After the eligibility criteria were applied, 2162 unique publications published between 1995 and 2025 were included in the final bibliometric analysis.

### 2.5 Validation of the Search Strategy

The search strategy was validated to reduce the possibility of false‐positive and false‐negative results. First, to minimise false‐positive results, the titles and abstracts of the top 100 cited publications were screened for topical relevance. Publications were considered relevant if they directly addressed leptin resistance, leptin responsiveness, leptin signalling impairment or leptin‐related metabolic dysfunction in the context of obesity, insulin resistance, diabetes, inflammation or cardiometabolic risk. The proportion of irrelevant or only partially related records was below 5%, supporting the specificity of the search strategy. Second, to assess the possibility of missing relevant documents, the retrieved publications were compared with the manually verified Scopus outputs of the top 20 most productive authors in the field. Agreement between the two approaches was assessed using the intraclass correlation coefficient. The strong agreement obtained (ICC = 0.964) supported the reliability of the search strategy in capturing relevant publications.

Overall, this validation step supported both the specificity and sensitivity of the search strategy. It reduced the risk of false‐positive publications through manual screening of highly cited records and the risk of false‐negative publications through comparison with the manually verified output of the most prolific authors. Similar validation approaches have been used in previous bibliometric studies to strengthen the reliability and reproducibility of search strategies [22, 23, 24, 25]. The precision of the search strategy and the risk of selection bias were further assessed by two independent researchers (S.A. and W.S.), who independently screened the titles and abstracts of the 100 most cited publications against the predefined eligibility criteria. Any disagreement was resolved through discussion and consensus. These independent checks applied specifically to the top‐100 relevance screening; the top‐20 author‐output comparison was a separate retrieval‐validation step.

### 2.6 Data Extraction

The retrieved records were exported from Scopus in CSV and RIS formats. The extracted information included publication year, document type, title, source title, affiliations, countries, abstracts, author keywords, indexed keywords, citation counts and references. A complete document‐level list of the included records, identified by DOI and/or Scopus EID, is provided in Supporting Information S1.

### 2.7 Bibliometric Indicators

Several bibliometric indicators were used to describe the global research landscape. These included annual publication growth, document types, most productive countries, most productive institutions, most active journals and most cited publications. The number and percentage of publications were calculated for each category. Citation analysis was used to identify influential publications in the field. The annual growth of publications was assessed to describe the development of research activity over time.

Productivity counts for countries and institutions used full counting: a publication was credited once to every represented country or institution. Consequently, country and institutional totals are not mutually exclusive and percentages may sum to more than 100%. Single‐country publications contained affiliations from one country, whereas multiple‐country or international collaborative publications contained affiliations from at least two countries. Total citations, citations per publication, corpus‐specific h‐indices and international collaboration percentages were recalculated from the supplied Scopus export. Country and author total link strengths were calculated within their respective co‐authorship networks. The 2025 CiteScore and SJR values were reported as source‐level journal metrics recorded separately from Scopus Sources and SCImago Journal Rank, respectively; they were not calculated from the study corpus.

### 2.8 Visualisation Analysis

Visualisation maps were generated using VOSviewer version 1.6.21. VOSviewer was used because it is widely applied in bibliometric research to construct and visualise scientific networks, including coauthorship, co‐occurrence and collaboration maps [28, 29]. In this study, VOSviewer was used to create international collaboration maps and term co‐occurrence maps on the basis of the titles and abstracts of the retrieved publications. For the international collaboration analysis, a minimum publication threshold was applied to identify countries with sufficient research output for inclusion in the network map. In the country collaboration map, each node represents a country, the node size reflects the number of publications and the thickness of the connecting lines reflects the strength of collaboration between countries. For the term co‐occurrence analysis, terms were extracted from the titles and abstracts of the retrieved publications. A minimum occurrence threshold was applied to identify frequently occurring terms for inclusion in the visualisation map. The size of each node reflected the frequency of term occurrence, whereas the distance and links between nodes reflected the strength of the association between terms. Network visualisation was used to identify major thematic clusters in the field. Overlay visualisation was used to display the temporal distribution of terms according to average publication year, where blue nodes represented earlier terms and yellow nodes represented more recent terms.

For reproducibility, the country co‐authorship analysis used a threshold of at least 20 publications, full counting and association‐strength normalisation. The title–abstract term map used a threshold of at least 50 occurrences (257 of 28,255 terms), binary counting and association‐strength normalisation.

### 2.9 Statistical Analysis

The retrieved data were exported to Microsoft Excel for cleaning and analysis. Frequencies and percentages were used to present the main bibliometric indicators, including document types, countries, institutions, journals and highly cited publications. The publication trajectory was summarised descriptively using annual counts, the compound annual growth rate from 1995 to 2025, the mean endpoint‐based annual increase calculated as the 2025 publication count minus the 1995 publication count divided by 30, the peak year and average output across broad periods. These measures were used for description rather than prediction. VOSviewer was used to generate and visualise the bibliometric maps.

### 2.10 Ethical Considerations

Ethical approval was not needed because the study was based on publicly available bibliographic data retrieved from Scopus and did not involve human participants, patient records or personal data.

## 3 Results

### 3.1 General Characteristics of the Retrieved Publications

**FIGURE 1:**
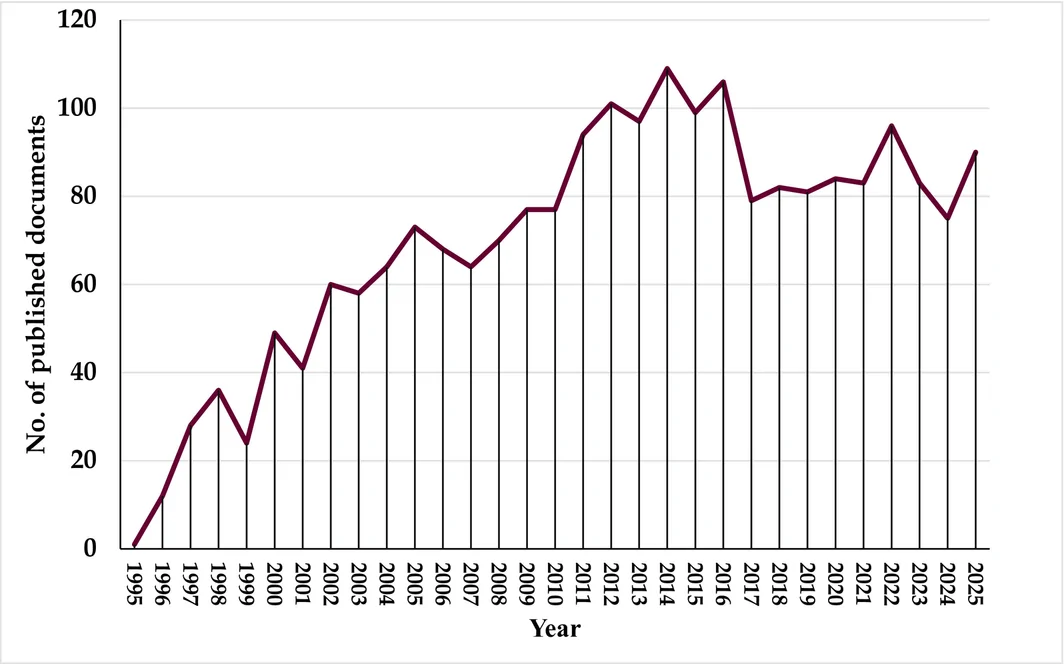
Annual growth of publications related to leptin resistance and obesity‐related metabolic dysfunction research from 1995 to 2025.

A total of 2162 publications on leptin resistance and obesity‐related metabolic dysfunction were retrieved from Scopus between 1995 and 2025. Output increased from one publication in 1995 to 91 in 2025, corresponding to a compound annual growth rate of 16.2% and a mean endpoint‐based increase of 3.0 publications per year. The trajectory was not steady: output peaked in 2014 (*n* = 109), fluctuated thereafter and averaged 87.2 publications annually during 2015–2025 compared with 37.3 during 1995–2004 (Figure 1). The lower recent counts, particularly for 2025, should be interpreted cautiously because delayed database indexing may affect the most recent year.

Among the retrieved documents, original articles represented the largest proportion (*n* = 1658; 76.69%), followed by reviews (*n* = 433; 20.03%). Other document types included conference papers (*n* = 41; 1.90%), short surveys (*n* = 21; 0.97%), notes (*n* = 7; 0.32%) and letters (*n* = 2; 0.09%) (Table 1).

**TABLE 1:** Distribution of publications by document type.

Document type	*n*	%
Article	1658	76.69
Review	433	20.03
Conference paper	41	1.90
Short survey	21	0.97
Note	7	0.32
Letter	2	0.09

Abbreviations: %, percentage of the 2162‐publication corpus; *n*, number of publications.

### 3.2 Most Productive Countries

Affiliation strings resolved to 76 countries, while 10 records did not contain a country label that could be parsed reliably. Using full counting, the United States ranked first, with 741 publications (34.27%), followed by China with 255 (11.79%), Japan with 132 (6.11%), Spain with 131 (6.06%), Germany with 124 (5.74%), Brazil with 119 (5.50%), the United Kingdom with 101 (4.67%), France with 87 (4.02%), Australia with 78 (3.61%) and Canada with 76 (3.52%) (Table 2). The United States also had the highest total citations (83,759) and total link strength (304), whereas the United Kingdom had the highest international collaboration percentage among the top 10 countries (57.43%).

**TABLE 2:** Productivity, citation impact and collaboration indicators for the top 10 countries in leptin resistance and obesity‐related metabolic dysfunction research.

Country	*n*	%	TC	CPP	SCP	MCP/ICP	ICP (%)	TLS
United States	741	34.27	83,759	113.04	519	222	29.96	304
China	255	11.79	7024	27.55	196	59	23.14	70
Japan	132	6.11	5855	44.36	99	33	25.00	40
Spain	131	6.06	7514	57.36	92	39	29.77	69
Germany	124	5.74	7393	59.62	69	55	44.35	93
Brazil	119	5.50	6786	57.03	89	30	25.21	37
United Kingdom	101	4.67	7323	72.50	43	58	57.43	93
France	87	4.02	6104	70.16	45	42	48.28	60
Australia	78	3.61	7784	99.79	41	37	47.44	51
Canada	76	3.52	7352	96.74	40	36	47.37	49

*Note:* Country output used full counting; therefore, a multicountry publication was credited once to every represented country.

Abbreviations: %, percentage of the total corpus; CPP, citations per publication; ICP (%), percentage of publications involving at least two countries; MCP/ICP, multiple‐country or international collaborative publications; *n*, number of publications; SCP, single‐country publications; TC, total citations; TLS, total link strength in the country co‐authorship network.

### 3.3 Most Productive Institutions

After targeted harmonisation of explicit affiliation variants, Harvard Medical School was the most productive institution, with 56 publications (2.59%), followed by INSERM with 49 (2.27%), Beth Israel Deaconess Medical Center with 48 (2.22%), Universidade de São Paulo with 37 (1.71%), UT Southwestern Medical Center and the University of Iowa with 33 each (1.53%), CIBEROBN, the University of Michigan and the University of Washington with 32 each (1.48%) and the University of Florida with 29 (1.34%) (Table 3). Harvard Medical School had the largest corpus‐specific h‐index (43), whereas Beth Israel Deaconess Medical Center had the highest total citations (12,239) and citations per publication (254.98). The aggregate label ‘VA Medical Center’ represented multiple named facilities and was therefore excluded from the institutional ranking rather than treated as one institution. Duplicate UT Southwestern labels were merged.

**TABLE 3:** Productivity and citation impact of the top institutions in leptin resistance and obesity‐related metabolic dysfunction research.

Institution	*n*	%	Corpus h‐index	TC	CPP
Harvard Medical School	56	2.59	43	11,974	213.82
INSERM	49	2.27	26	3391	69.20
Beth Israel Deaconess Medical Center	48	2.22	43	12,239	254.98
Universidade de São Paulo	37	1.71	20	2183	59.00
UT Southwestern Medical Center	33	1.53	25	3209	97.24
University of Iowa	33	1.53	26	4060	123.03
CIBEROBN	32	1.48	21	3103	96.97
University of Michigan	32	1.48	24	3905	122.03
University of Washington	32	1.48	27	5014	156.69
University of Florida	29	1.34	22	2015	69.48

*Note:* The corpus h‐index is calculated only from publications included in this study.

Abbreviations: %, percentage of the total corpus; CPP, citations per publication; *n*, number of publications; TC, total citations.

### 3.4 Most Productive Journals

Endocrinology was the most active journal, with 73 publications (3.38%), followed by Diabetes with 58 (2.68%) and the International Journal of Obesity with 47 (2.17%). PLOS One ranked fourth with 42 publications (1.94%), followed by the American Journal of Physiology—Regulatory, Integrative and Comparative Physiology with 36 (1.67%), International Journal of Molecular Sciences with 35 (1.62%), American Journal of Physiology—Endocrinology and Metabolism with 34 (1.57%), Cell Metabolism with 30 (1.39%), Molecular Metabolism with 29 (1.34%) and Frontiers in Endocrinology with 27 (1.25%) (Table 4). Diabetes had the highest total citations (9849), Cell Metabolism had the highest citations per publication (178.60) and Endocrinology had the highest corpus‐specific h‐index (47).

**TABLE 4:** Productivity, citation impact and source indicators of the top 10 journals publishing research on leptin resistance and obesity‐related metabolic dysfunction.

Journal	*n*	%	TC	CPP	CiteScore (2025)	SJR (2025)	Corpus h‐index
Endocrinology	73	3.38	7733	105.93	6.8	1.302	47
Diabetes	58	2.68	9849	169.81	10.8	2.613	43
International Journal of Obesity	47	2.17	4119	87.64	10.8	1.572	34
PLoS ONE	42	1.94	2899	69.02	4.8	0.726	25
American Journal of Physiology‐Regulatory Integrative and Comparative Physiology	36	1.67	2552	70.89	4.5	0.902	26
International Journal of Molecular Sciences	35	1.62	1556	44.46	10	1.316	20
American Journal of Physiology‐Endocrinology and Metabolism	34	1.57	3794	111.59	5.9	1.505	27
Cell Metabolism	30	1.39	5358	178.60	54.1	15.978	26
Molecular Metabolism	29	1.34	974	33.59	11.9	2.918	18
Frontiers in Endocrinology	27	1.25	2030	75.19	9.1	1.566	17

*Note:* The corpus h‐index is restricted to publications included in this study. CiteScore and SJR are 2025 source‐level journal metrics recorded separately from Scopus Sources and SCImago Journal Rank, respectively; they are not derived from the 2162‐publication study corpus.

Abbreviations: %, percentage of the total corpus; CPP, citations per publication; *n*, number of publications; SJR, SCImago Journal Rank; TC, total citations.

### 3.5 Most Cited Publications

The citation analysis identified the most highly cited publications within the retrieved corpus. The most cited article was the Nature publication by Cowley et al. on leptin activation of anorexigenic POMC neurons (2052 citations), followed by the Nature Medicine article by Frederich et al. on diet‐induced resistance to leptin action (1533 citations) and the Endocrine Reviews review by Kalra et al. on hypothalamic regulation of body weight (1457 citations). Table 5 additionally reports citations per year and a year‐normalised citation ratio calculated against the mean citation count of all corpus publications from the same year.

**TABLE 5:** Top 20 cited publications in leptin resistance and obesity‐related metabolic dysfunction research, with citation rate and normalised‐impact fields.

Authors	Title	Year	Source title	Cited by	Citations/year^a^	Year‐normalised citation ratio^b^
Cowley M.A. et al.	Leptin activates anorexigenic POMC neurons through a neural network in the arcuate nucleus	2001	Nature	2052	82.1	16.77
Frederich R.C. et al.	Leptin levels reflect body lipid content in mice: Evidence for diet‐induced resistance to leptin action	1995	Nature Medicine	1533	49.5	1.00
Kalra S.P. et al.	Interacting appetite‐regulating pathways in the hypothalamic regulation of body weight	1999	Endocrine Reviews	1457	54.0	8.90
Galic S. et al.	Adipose tissue as an endocrine organ	2010	Molecular and Cellular Endocrinology	1430	89.4	15.76
Caro J.F. et al.	Decreased cerebrospinal‐fluid/serum leptin ratio in obesity: A possible mechanism for leptin resistance	1996	Lancet	1115	37.2	3.14
Coskun T. et al.	Fibroblast growth factor 21 corrects obesity in mice	2008	Endocrinology	971	53.9	8.76
Everard A. et al.	Responses of gut microbiota and glucose and lipid metabolism to prebiotics in genetic obese and diet‐induced leptin‐resistant mice	2011	Diabetes	968	64.5	11.66
Schwartz M.W. et al.	Cerebrospinal fluid leptin levels: Relationship to plasma levels and to adiposity in humans	1996	Nature Medicine	961	32.0	2.70
Halaas J.L. et al.	Physiological response to long‐term peripheral and central leptin infusion in lean and obese mice	1997	Proceedings of the National Academy of Sciences of the United States of America	950	32.8	4.71
Obradovic M. et al.	Leptin and Obesity: Role and Clinical Implication	2021	Frontiers in Endocrinology	947	189.4	24.90
Milanski M. et al.	Saturated fatty acids produce an inflammatory response predominantly through the activation of TLR4 signalling in hypothalamus: Implications for the pathogenesis of obesity	2009	Journal of Neuroscience	934	54.9	8.49
Bjørbæk C. et al.	Identification of SOCS‐3 as a potential mediator of central leptin resistance	1998	Molecular Cell	897	32.0	7.44
Schwartz M.W. et al.	Specificity of leptin action on elevated blood glucose levels and hypothalamic neuropeptide Y gene expression in ob/ob mice	1996	Diabetes	881	29.4	2.48
Schwartz M.W. et al.	Leptin increases hypothalamic pro‐opiomelanocortin mRNA expression in the rostral arcuate nucleus	1997	Diabetes	812	28.0	4.02
Ozcan L. et al.	Endoplasmic Reticulum Stress Plays a Central Role in Development of Leptin Resistance	2009	Cell Metabolism	788	46.4	7.16
Cani P.D. et al.	Involvement of gut microbiota in the development of low‐grade inflammation and Type 2 diabetes associated with obesity	2012	Gut Microbes	768	54.9	11.66
El‐Haschimi K. et al.	Two defects contribute to hypothalamic leptin resistance in mice with diet‐induced obesity	2000	Journal of Clinical Investigation	762	29.3	8.26
Zabolotny J.M. et al.	PTP1B regulates leptin signal transduction in vivo	2002	Developmental Cell	761	31.7	7.68
Myers M.G. et al.	Obesity and leptin resistance: Distinguishing cause from effect	2010	Trends in Endocrinology and Metabolism	731	45.7	8.06
Rahmouni K. et al.	Obesity‐associated hypertension: New insights into mechanisms	2005	Hypertension	728	34.7	10.90

*Note:* Cited by indicates the total Scopus citation count as of August 9, 2026.

^a^ Citations per year were calculated as total citations divided by the number of elapsed years through 2026.

^b^ The year‐normalised citation ratio is the publication citation count divided by the mean citation count of all corpus publications published in the same year.

The topics represented among these highly cited publications spanned three connected areas: central leptin signalling and appetite regulation, mechanisms of leptin resistance in obesity and wider metabolic and inflammatory consequences of adipose tissue dysfunction. Highly cited studies on the gut microbiota, inflammatory signalling, endoplasmic reticulum stress and hypertension show that the retrieved corpus also includes work linking leptin resistance with broader obesity‐related metabolic dysfunction.

### 3.6 Boundary Sensitivity Analysis

The narrower Scopus query, which excluded ‘leptin sensitivity’ and ‘leptin responsiveness,’ retrieved 1881 unique publications. All 1881 EIDs were present in the primary expanded corpus. Thus, the narrower query retained 87.00% of the primary corpus, whereas 281 publications (13.00%) represented the increment attributable to the two broader expressions (Table 6).

**TABLE 6:** Boundary sensitivity analysis of the search strategy.

Search specification	Records (*n*)	% of primary corpus	Interpretation
Primary expanded query	2162	100.00	Reference corpus
Narrow explicit‐resistance query	1881	87.00	Actual Scopus rerun
Increment attributable to sensitivity/responsiveness expressions	281	13.00	Expanded minus narrower query
Narrow‐query records outside the primary corpus	0	0.00	All EIDs matched the primary corpus

*Note:* The primary expanded query included ‘leptin sensitivity’ and ‘leptin responsiveness.’ The narrower query removed only these two expressions while retaining all other terms and limits. The narrower search was rerun in Scopus on August 10, 2026, and all 1881 retrieved EIDs were present in the primary corpus.

Abbreviations: %, percentage of the 2162‐record primary corpus; EID, Scopus electronic identifier; *n*, number of records.

### 3.7 Most Productive Authors

Author records were disambiguated using Scopus Author IDs. Lisboa P.C. was the most productive author (*n* = 33), followed by Moura E.G. (*n* = 31) and Rahmouni K. (*n* = 26). Among the top 10 authors, Rahmouni K. had the highest total citations (3362) and corpus‐specific h‐index (22), whereas Donato Jr. J. had the highest international collaboration percentage (38.89%). Lisboa P.C. had the highest author co‐authorship total link strength (221) (Table 7).

**TABLE 7:** Top 10 authors by publication output and citation impact.

Author	n	TC	CPP	Corpus h‐index	ICP (%)	TLS
Lisboa, P.C.	33	1138	34.48	20	3.03	221
Moura, E.G.	31	1114	35.94	20	3.23	207
Rahmouni, Kamal	26	3362	129.31	22	7.69	84
Scarpace, Philip J.	24	1960	81.67	20	0.00	78
De Oliveira, Elaine	22	628	28.55	16	0.00	145
Banks, William A.	19	2434	128.11	19	31.58	67
Donato Jr., Jose	18	1001	55.61	14	38.89	106
Hosoi, Toru	18	450	25.00	9	16.67	73
Gonzalez‐Bulnes, A.	18	405	22.50	11	16.67	120
Ozawa, Koichiro	17	449	26.41	9	17.65	73

*Note:* The corpus h‐index is restricted to publications included in this study. Authors were disambiguated using Scopus Author IDs.

Abbreviations: CPP, citations per publication; ICP (%), percentage of publications involving at least two countries; *n*, number of publications; TC, total citations; TLS, total link strength in the author co‐authorship network.

### 3.8 International Collaboration Network

**FIGURE 2:**
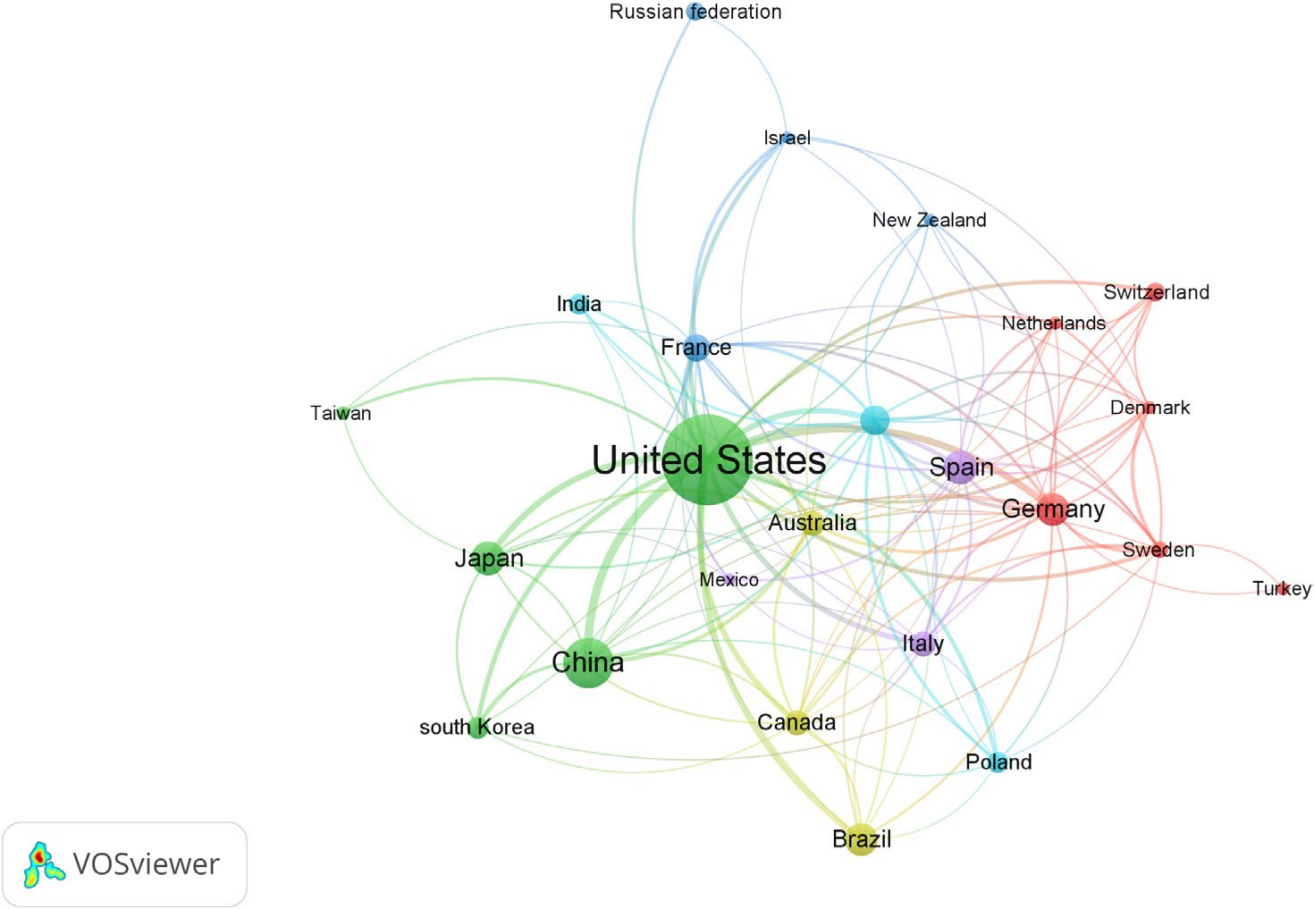
International collaboration network among countries in leptin resistance and obesity‐related metabolic dysfunction research. Countries with at least 20 publications were included (24 of 76 countries resolved from affiliation strings). Node size represents publication output, link thickness represents collaboration strength and colour identifies the VOSviewer cluster. Full counting and association‐strength normalisation were used.

Of the 76 countries resolved from affiliation strings, 24 met the minimum threshold of 20 publications and were included in the network. The United States had the largest node and strongest total link strength, followed by several European, Asian and other high‐output countries. Because countries below the threshold were not displayed, the network describes collaboration among higher‐output countries rather than the absence of collaboration elsewhere (Figure 2).

The United States had the largest node in the network and showed collaborative links with several countries, including China, Japan, Canada, Germany, France, Spain, Australia, Italy and Brazil. China also had a large node and showed collaborative links with the United States, Japan, South Korea and Canada. Network visualisation revealed several clusters of collaborating countries. One cluster included the United States, China, Japan, South Korea and Taiwan. Another cluster included Germany, Switzerland, Denmark, Sweden, the Netherlands and Turkey. A third cluster included France, Israel, New Zealand, India and the Russian Federation. Other countries, such as Canada, Brazil, Australia, Spain, Italy, Poland and Mexico, were also connected within the collaboration network.

### 3.9 Network Visualisation of Term Co‐Occurrence

**FIGURE 3:**
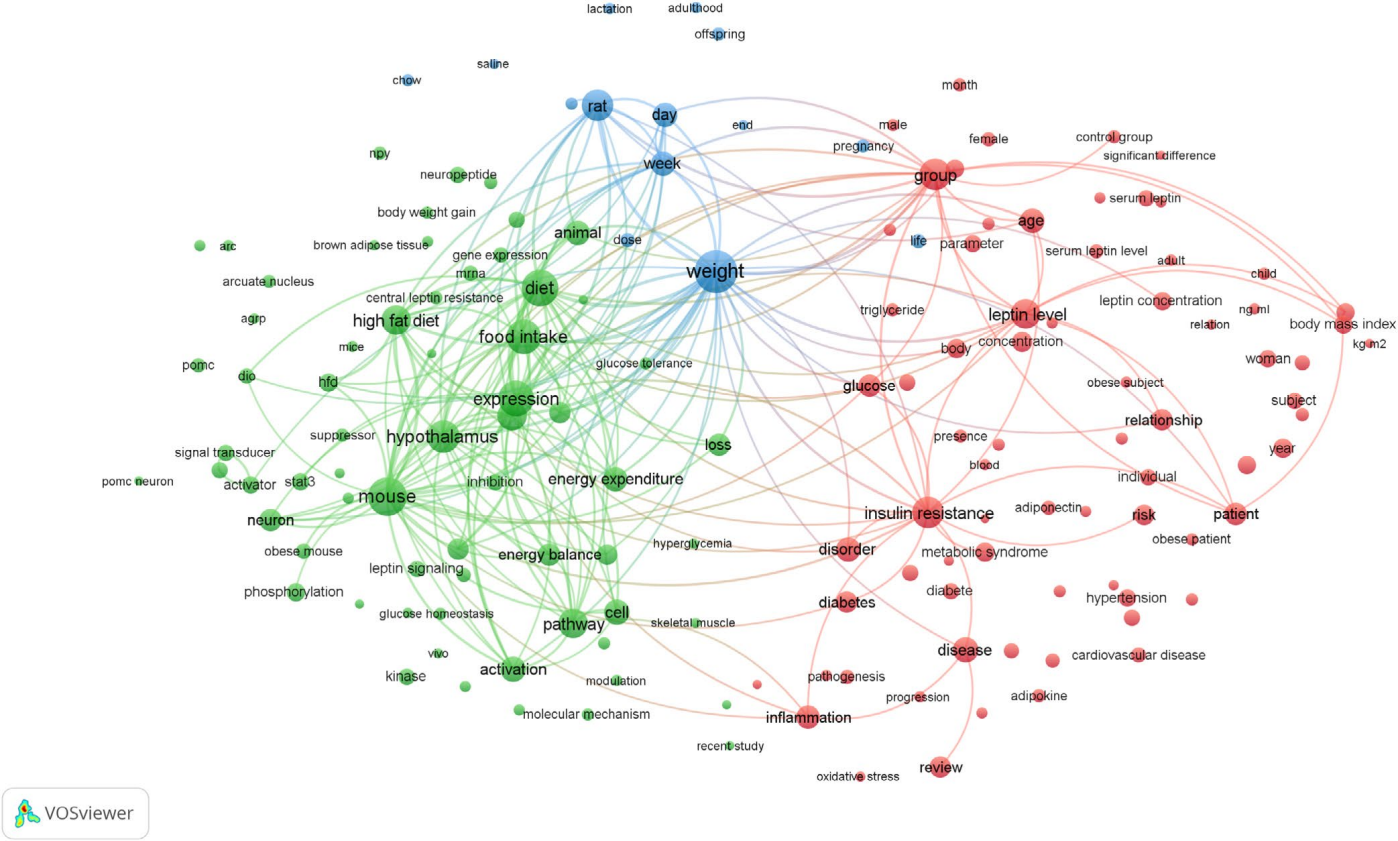
Network visualisation of title–abstract term co‐occurrence in leptin resistance and obesity‐related metabolic dysfunction research. Terms occurring at least 50 times were included (257 of 28,255 terms). Node size represents occurrence frequency, link thickness represents co‐occurrence strength, shorter distance indicates a stronger relationship and colour identifies the thematic cluster assigned by VOSviewer.

A term co‐occurrence analysis was performed using VOSviewer version 1.6.21 on the basis of the titles and abstracts of the 2162 retrieved publications. A minimum occurrence threshold of 50 was applied. Of the 28,255 terms identified, 257 met the threshold and were included in the visualisation map. Network visualisation revealed three main clusters (Figure 3). The first cluster included terms related to clinical metabolic dysfunction and cardiometabolic risk, such as leptin level, insulin resistance, glucose, diabetes, metabolic syndrome, body mass index, inflammation, adiponectin, hypertension, cardiovascular disease, risk, patient, serum leptin and leptin concentration. The second cluster included terms related to hypothalamic leptin signalling, appetite control and energy homeostasis, such as hypothalamus, food intake, body weight, energy expenditure, neuropeptide, melanocortin, POMC neuron, arcuate nucleus, leptin receptor, diet‐induced obesity and STAT3. The third cluster included terms related to body weight regulation and developmental and experimental models, such as weight, rat, animal, glucose tolerance, pregnancy, offspring, lactation, adulthood, saline and chow.

### 3.10 Overlay Visualisation of Term Co‐Occurrence

An overlay visualisation map of term co‐occurrence was generated using VOSviewer version 1.6.21 to show the temporal distribution of research terms in the field of leptin resistance and obesity‐related metabolic dysfunction. In this map, the colour of each node represents the average publication year of the term.

**FIGURE 4:**
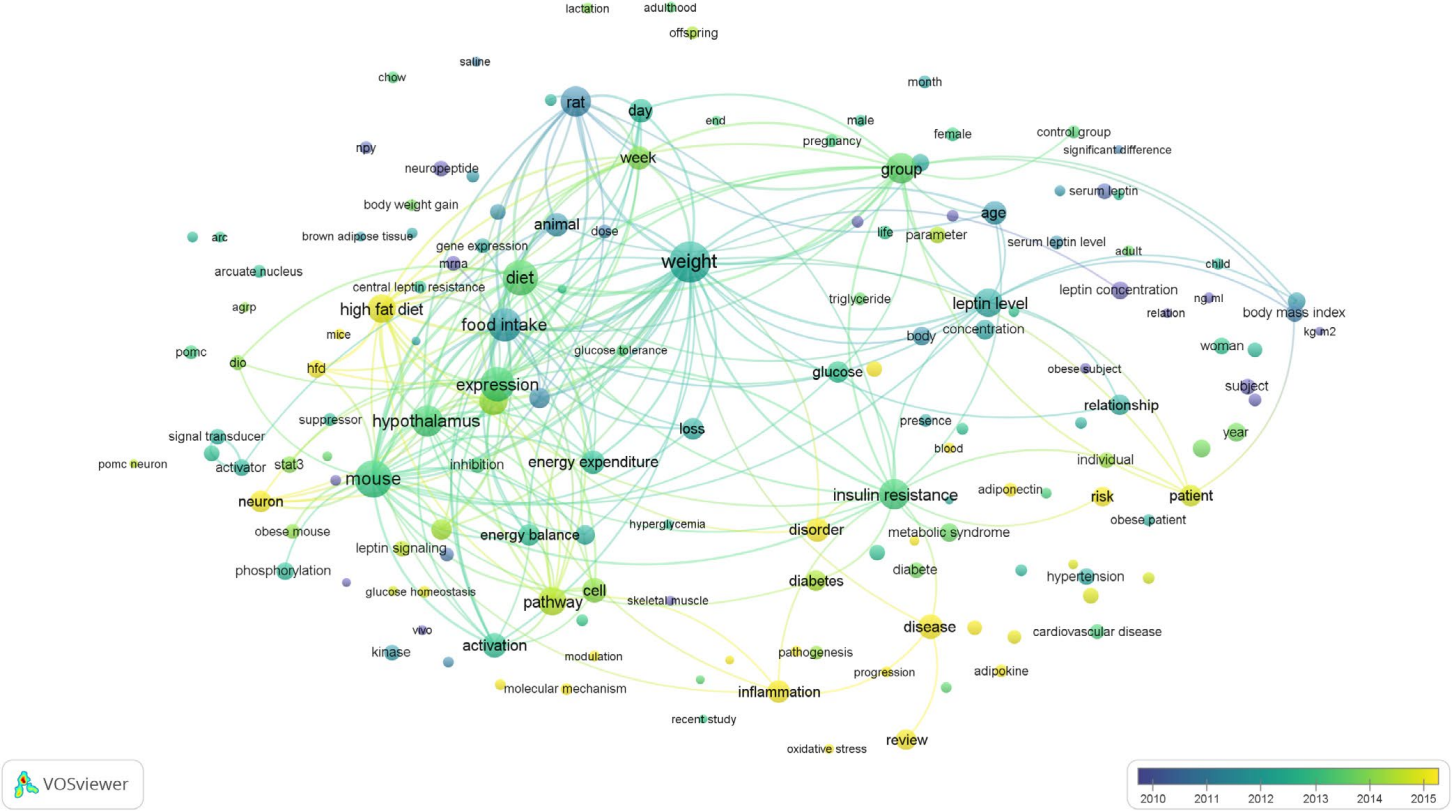
Overlay visualisation of title–abstract term co‐occurrence in leptin resistance and obesity‐related metabolic dysfunction research. Node size represents occurrence frequency and overlay colour represents average publication year, from earlier terms in blue through intermediate terms in green to more recent terms in yellow. Colours describe terminology timing and do not establish a causal thematic transition.

The blue nodes represent earlier terms, the green nodes represent terms appearing in the intermediate period and the yellow nodes represent more recent terms (Figure 4). Earlier terms included rat, age, serum leptin, serum leptin level, leptin concentration, body mass index, neuropeptide and mRNA.

Terms appearing in the intermediate period included weight, diet, food intake, hypothalamus, mouse, expression, group, leptin level, glucose and insulin resistance. More recent terms included inflammation, disease, diabetes, oxidative stress, pathogenesis, progression, risk, patient, individual, hypertension, cardiovascular disease, adipokine, adiponectin, high‐fat diet, HFD, neuron, molecular mechanism, glucose homeostasis and pathway.

## 4 Discussion

This bibliometric and visualisation analysis mapped 2162 Scopus publications on leptin resistance and obesity‐related metabolic dysfunction from 1995 to 2025. Publication activity increased substantially over the full period but peaked in 2014 and remained broadly stable thereafter. Productivity, citation impact and collaboration were concentrated among a limited group of countries, institutions, journals and authors. The United States led country output and collaboration, Harvard Medical School led institutional output after targeted affiliation harmonisation and Endocrinology was the most active journal. The term maps linked foundational leptin signalling and energy‐homeostasis research with later‐published inflammatory, molecular and cardiometabolic terminology. Because overlay timing can also reflect lexical fashion, indexing and changes in disease nomenclature, these patterns should be interpreted as descriptive evidence of vocabulary broadening rather than proof of a causal transition.

The increase in publications shows that leptin resistance has received sustained research attention in obesity and metabolic disease. This pattern is consistent with the broader view of obesity as a complex condition involving adipose tissue dysfunction, neuroendocrine changes, inflammation, insulin resistance and cardiometabolic complications [1, 2, 30]. Early publications were mainly concerned with leptin levels, adiposity and appetite regulation after the discovery of leptin [5, 6]. Recent studies have focused on leptin signalling defects, hypothalamic inflammation, endoplasmic reticulum stress, insulin resistance, the gut microbiota, adipokines, cardiovascular risk and metabolic disease progression [10, 11, 12, 13, 14, 15, 16, 17, 18, 19, 20, 21, 31, 32, 33, 34, 35]. Taken together, these temporal patterns are consistent with broader attention to obesity as an endocrine, inflammatory, neuronal and cardiometabolic disorder, but they do not establish a single directional shift in research practice.

The high proportion of original articles indicates that leptin resistance research is still driven mainly by experimental, clinical and translational studies. This is expected because leptin resistance remains a complex biological condition without a single accepted clinical definition. Several mechanisms, including altered leptin concentration, impaired leptin transport, defective receptor signalling, hypothalamic inflammation and peripheral metabolic disturbances, have been proposed [3, 4, 21, 30, 31, 32]. The relatively high number of reviews also suggests that the field has reached a stage where accumulated evidence requires synthesis and clearer conceptual organisation.

Country analysis revealed that research on leptin resistance and obesity‐related metabolic dysfunction has been concentrated mainly in a few countries. The United States ranked first in terms of publication output and had the strongest position in the international collaboration network. China ranked second in publication output. Other productive countries were Japan, Spain, Germany, Brazil, the United Kingdom, France, Australia and Canada. Brazil was among the leading contributing countries, but the bulk of the research output was still dominated by countries with strong biomedical research capacity. These findings should be interpreted cautiously because publication output does not directly measure disease burden or research need. Although obesity, Type 2 diabetes and cardiometabolic diseases are major global concerns, the analysis did not test whether additional collaboration would change research quality or health outcomes [1, 2, 30].

The institutional analysis showed concentration among leading academic and medical centres. After excluding the non‐specific ‘VA Medical Center’ aggregate and merging UT Southwestern variants, Harvard Medical School led output, while Beth Israel Deaconess Medical Center had the highest citation total and average among the top 10 institutions. These indicators describe productivity and citation influence within this corpus; they do not measure research quality or institutional performance outside the defined search boundary.

Journal analysis showed that leptin resistance research was published across several related fields. The most prolific journals were *Endocrinology*, *Diabetes*, *International Journal of Obesity, PLOS One, American Journal of Physiology, International Journal of Molecular Sciences, Cell Metabolism, Molecular Metabolism* and *Frontiers in Endocrinology*. This pattern reflects the multidisciplinary nature of leptin resistance research, which is related to endocrinology, obesity, physiology, neuroscience, molecular biology, diabetes and cardiometabolic medicine [3, 4, 19, 20, 30, 33]. The mixture of basic‐science and clinically oriented journals is consistent with coverage across experimental, molecular, translational and clinical topics, including metabolic disease, inflammation, cardiovascular risk and patient‐related outcomes.

The most cited publications reflected the intellectual base of leptin resistance research. The top‐cited studies focused mainly on hypothalamic leptin signalling, diet‐induced leptin resistance, the cerebrospinal fluid/serum leptin ratio, PTP1B, endoplasmic reticulum stress, hypothalamic inflammation and defects in leptin responsiveness [5, 7, 10, 11, 12, 13, 14]. These publications help establish central leptin signalling and impaired leptin activity as major research directions in obesity. More recent reviews and experimental studies have further linked leptin resistance with impaired leptin transport, LepRb signalling, SOCS3, PTP1B, ER stress and inflammatory pathways [21, 30, 31, 32, 33, 34, 35, 36]. Therefore, the citation profile shows that the field has been built mainly around central mechanisms of leptin resistance and their role in obesity‐related metabolic dysfunction.

Several highly cited publications were also related to the wider field of obesity‐associated metabolic dysfunction. These included studies on adipose tissue as an endocrine organ, gut microbiota and glucose–lipid metabolism, low‐grade inflammation and Type 2 diabetes and obesity‐associated hypertension [15, 16, 17, 18]. These publications show that the highly cited corpus also includes studies beyond leptin signalling alone. The cited literature links leptin resistance with adipose tissue dysfunction, systemic inflammation, metabolic disease and cardiovascular risk. Recent reviews have also connected leptin resistance with obesity, Type 2 diabetes, cardiovascular disease, insulin‐related pathways, endothelial dysfunction, oxidative stress and therapeutic challenges [19, 20, 37, 38]. These findings support the study of leptin resistance within the broader context of obesity‐related metabolic dysfunction.

The country collaboration map revealed that the international research network was connected but concentrated around a limited number of productive countries. The United States had the largest node and the strongest collaborative links with China, Japan, Canada, Germany, France, Spain, Australia, Italy and Brazil. China also clearly collaborated with the United States and several Asian countries. European countries formed another important collaboration network, while Brazil, Canada, Australia, Spain, Italy, Poland and Mexico contributed to different parts of the map. These findings indicate that collaboration in leptin resistance research is active but still dominated by countries with high research output. Because the network map excluded countries below the 20‐publication threshold, it characterises collaboration among higher‐output countries and should not be used to conclude that excluded countries had no international links. Future analyses using lower thresholds could better describe participation by underrepresented regions.

The term co‐occurrence analysis identified three main research themes. The first theme was ‘clinical metabolic dysfunction and cardiometabolic risk.’ This theme included terms related to leptin level, insulin resistance, glucose, diabetes, metabolic syndrome, body mass index, inflammation, adiponectin, hypertension, cardiovascular disease, serum leptin and leptin concentration. These terms show that leptin resistance has been widely studied in relation to impaired glucose metabolism, metabolic syndrome, inflammatory markers and cardiometabolic outcomes [16, 17, 18, 19, 20, 37, 38, 39]. This cluster captures clinically oriented questions concerning obesity‐associated metabolic and cardiovascular risk rather than demonstrating a temporal shift in the field.

The second theme was ‘hypothalamic leptin signalling, appetite control, and energy homeostasis.’ This theme included experimental and mechanistic terms such as hypothalamus, mouse, food intake, high‐fat diet, energy expenditure, energy balance, leptin signalling, glucose homeostasis, gene expression, arcuate nucleus, POMC, AgRP, NPY and brown adipose tissue. This cluster represents the basic biological foundation of leptin resistance research [7, 8, 9, 10, 11, 12, 13, 14, 21, 30, 31, 32, 33, 34, 35, 36]. Animal models, particularly high‐fat diet models, are important for studying impaired leptin signalling and its effects on appetite, body weight, thermogenesis and glucose regulation.

The third theme was ‘body weight regulation and developmental programming.’ This theme included terms such as weight, rat, animal, glucose tolerance, pregnancy, offspring, lactation, adulthood, saline and chow. The presence of pregnancy, lactation, offspring and adulthood suggests that some of the literature has focused on early‐life exposure, maternal metabolic status and nutritional programming in relation to leptin resistance and later metabolic dysfunction [40, 41]. These findings indicate that developmental and experimental models remain important for understanding body weight regulation and long‐term cardiometabolic risk.

The overlay visualisation showed differences in the average publication year of terms used in the field. Earlier terms were related mainly to leptin measurement, serum leptin concentration, body mass index, rat models and the expression of neuropeptides and mRNAs. These terms were more common in earlier publications and are consistent with work defining leptin biology and its relationship with adiposity and central appetite regulation [5, 6, 7, 8, 9]. Intermediate terms such as weight, diet, food intake, hypothalamus, mouse, leptin level, glucose and insulin resistance were associated with an intermediate average publication year and included mechanistic studies linking leptin resistance to energy balance and glucose metabolism [10, 11, 12, 13, 14, 21, 30, 31, 32, 33, 34, 35, 36]. More recent terms, including inflammation, disease, diabetes, oxidative stress, pathogenesis, progression, risk, patient, cardiovascular disease, adipokine, high‐fat diet, molecular mechanism, glucose homeostasis and pathway, had later average publication years and may reflect greater attention to clinical risk, inflammatory pathways and molecular mechanisms of obesity‐related metabolic disease [19, 20, 37, 38, 42].

The overlay visualisation is consistent with a broadening of the vocabulary used in leptin resistance research towards clinical and translational topics. Recent terminology concerning inflammation, insulin resistance, cardiovascular risk, endothelial dysfunction, patient heterogeneity and treatment response may reflect increasing attention to the role of leptin resistance in obesity‐related metabolic complications [19, 20, 37, 38, 43]. These patterns are exploratory. They may reflect changes in terminology, indexing or disease nomenclature, including the replacement of NAFLD by MAFLD or MASLD, as well as changes in research emphasis; therefore, the overlay map cannot by itself establish a substantive or causal transition.

The results also suggest several directions for future research. The need for a clearer and more useful definition of leptin resistance is evident, and more clinical studies are needed to provide this, as no widely accepted clinical measure is available [30, 32, 38]. Future research should also consider exploring leptin resistance in individuals with other phenotypes of obesity and related disorders, such as visceral obesity, diabetes, fatty liver disease, cardiovascular disease and metabolically healthy obesity [19, 20, 37, 38]. Further translational studies are needed to link the mechanisms observed in animal models to human metabolic outcomes [21, 30, 31, 32, 33, 34, 35, 36]. The therapeutic potential of leptin resistance warrants further study, especially with respect to lifestyle modification, antiobesity drugs, bariatric surgery, anti‐inflammatory interventions and other strategies that may increase leptin sensitivity [3, 19, 20, 21, 43]. Future studies could examine whether broader geographic participation changes the populations, settings and research questions represented in this literature.

### 4.1 Strengths and Limitations

This study has several strengths. It is based on a large Scopus dataset covering publications from 1995 to 2025 and combines descriptive bibliometric indicators with visualisation analysis. The use of country collaboration mapping, term co‐occurrence analysis and overlay visualisation allowed a broad assessment of publication trends, collaboration patterns, research hotspots and thematic evolution. In addition, the search strategy was validated by comparing the retrieved dataset with the manually verified Scopus output of the top 20 most productive authors. The strong agreement observed using the intraclass correlation coefficient supports the reliability of the search strategy and reduces the possibility of missing major relevant publications. Similar validation approaches have been used in previous bibliometric studies to improve the reliability and reproducibility of search strategies [22, 23, 24, 25].

This study has several limitations. First, the analysis was conducted using only the Scopus database. While Scopus has wide coverage of peer‐reviewed literature, it is possible that some relevant publications indexed in other databases, such as Web of Science, PubMed or Embase, were missed. Second, the number of citations may be affected by the age of the publication, the visibility of the journal, the size of the field, self‐citation and the coverage of the database. Citation counts should thus not be read as a direct measure of study quality or clinical importance. Third, bibliometric findings depend on the accuracy and consistency of the metadata indexed in the database. Variations in author names, the inconsistent use of initials or full names, spelling errors, name changes and the presence of common names may divide one researcher's publications across multiple profiles or incorrectly combine the work of different researchers. Similar difficulties affect institutional data because affiliations may be reported using abbreviations, translated names, departmental addresses, former institutional names or different spellings. Changes in institutional affiliation over time may introduce further uncertainty. Although Scopus Author IDs and targeted harmonisation of explicit institutional variants were used in this study, these procedures cannot eliminate all disambiguation errors. Therefore, author‐ and institution‐level productivity, citation indicators and collaboration networks should be interpreted cautiously because residual misclassification may result in the underestimation or overestimation of research contributions [44, 45]. Fourth, the analysis of co‐occurrence was obtained from titles and abstracts, which may not include all the concepts discussed in the full text. Additionally, the thresholds used in VOSviewer could ignore some less frequent but possibly emerging topics. Fifth, Scopus records and citation counts are updated over time and the findings reflect the status of the database at the time of data extraction. Lastly, although no language restriction was applied, the search strategy was based mainly on English terms. Therefore, relevant publications with non‐English titles or abstracts may have been missed. In addition, the inclusion of broader terms such as ‘leptin sensitivity’ and ‘leptin responsiveness’ may have retrieved some studies that were not primarily focused on leptin resistance; however, the validation step was used to reduce this risk.

Although the query used English search expressions, the retrieved corpus included 164 non‐English‐language records (7.59%). This proportion describes the language composition of the retrieved set but cannot quantify records missed because they lacked English titles, abstracts or keywords. The overlay map should also be interpreted as a map of changing vocabulary rather than direct evidence of a transition in scientific practice. Lexical fashion and nomenclature changes, such as NAFLD, MAFLD and MASLD, may alter average publication years independently of substantive changes in research focus.

## 5 Conclusions

This bibliometric and visualisation analysis showed that research on leptin resistance and obesity‐related metabolic dysfunction increased markedly from 1995 to 2025, although annual output fluctuated and remained broadly stable after the 2014 peak. Earlier and later term profiles were consistent with a broadening from foundational leptin signalling, hypothalamic appetite regulation and animal models towards inflammation, metabolic disease progression, cardiometabolic risk and patient‐oriented terminology, while remaining vulnerable to changes in nomenclature and indexing. Research productivity remained concentrated among a limited number of countries and institutions, while country‐level collaboration was concentrated among higher‐output countries, with the United States occupying the most central position in the country network. The mapped gaps support further study of clinical phenotyping, translational mechanisms, therapeutic strategies and geographic participation, but these priorities should be tested using study‐level evidence rather than inferred from bibliometric patterns alone.

## Author Contributions

**Sa'ed. H. Zyoud:** conceptualization, investigation, writing – original draft, methodology, validation, visualization, writing – review and editing, software, formal analysis, data curation, supervision, resources, project administration.

## Funding

The author has nothing to report.

## Ethics Statement

This study was based on publicly available bibliographic data retrieved from the Scopus database. No human participants, patient records, biological samples or individual‐level personal data were used.

## Consent

The author has nothing to report.

## Conflicts of Interest

The author declares no conflicts of interest.

## Supporting information


**Supporting Information S1:** Document‐level list of publications included in the bibliometric analysis. This file contains the bibliographic identifiers for all 2162 publications included in the final Scopus dataset, including DOI and/or Scopus EID where available.

## Data Availability

The data used in this study were retrieved from the Scopus database. The datasets generated and analysed during the current study are available from the corresponding author upon reasonable request and are subject to database access and licensing restrictions. Supporting Information S1 provides the DOI and/or Scopus EID document list for all 2162 included records, together with basic bibliographic identifiers.
